# A Rare Natural Benzo[*k*,*l*]xanthene as a Turn-Off Fluorescent Sensor for Cu^2+^ Ion

**DOI:** 10.3390/ijms21186933

**Published:** 2020-09-21

**Authors:** Giuseppe Floresta, Nunzio Cardullo, Carmela Spatafora, Antonio Rescifina, Corrado Tringali

**Affiliations:** 1 Department of Drug Sciences, University of Catania, Viale A. Doria 6, 95125 Catania, Italy; giuseppe.floresta@unict.it; 2 Department of Chemical Sciences, University of Catania, Viale A. Doria 6, 95125 Catania, Italy; cspatafo@unict.it (C.S.); ctringali@unict.it (C.T.)

**Keywords:** polyphenols, lignans, fluorescent sensor, natural-derived ligand

## Abstract

Rapid and efficient analyses of copper ions are crucial to providing key information for Cu^2+^ in living cells because of their biological importance. In this study, we reported one new turn-off fluorescent sensor for Cu^2+^ with a benzo[*k*,*l*]xanthene core, which served as an efficient cation sensor for copper ion over a wide range of other cations (Na^+^, K^+^, Ag^+^, Hg^2+^, Cd^2+^, Co^2+^, Ni^2+^, Zn^2+^, Mg^2+^, and Fe^3+^) owing to the catechol group in the aromatic core. The sensor showed selectivity for Cu^2+^ over other ions; the log*K*_β_ for Cu^2+^ binding to compound **1** had a value of 13.265. In the presence of Cu^2+^, sensor **1** provided significant fluorescence decrement; Co^2+^, and Ni^2+^ caused a fluorescence decrement when employed at a higher concentration than Cu^2+^, while Na^+^, K^+^, Hg^2+^, Cd^2+^, Zn^2+^, and Mg^2+^ metal ions produced only minor changes in fluorescence intensity. Fluorescence experiments demonstrate that compound **1** may have an application as a fluorescent probe for detecting Cu^2+^ with a limit of detection of 0.574 µM.

## 1. Introduction

The design and synthesis of fluorescent sensors with high selectivity for cations have recently received considerable attention because of the fundamental role of cations in living systems. Indeed, some ions such as sodium, potassium, magnesium, and calcium are involved in many biological processes, and the effective control of their levels is of fundamental importance in medicine.

The development of sensitive and effective fluorescent sensors for heavy transition metal ions is also of current interest. These metals are used in many different industrial processes, and there is a high risk of their release into the environment; consequently, the search for methods to monitor and reveal these ions is of great relevance.

Among the various ions of this type, copper is worth particular attention; in fact, it has a catalytic role as a cofactor for a variety of metalloenzymes, including superoxide dismutase, cytochrome c, oxidase, and tyrosinase. However, in conditions that favor accumulation, copper can cause neurodegenerative diseases [1] (there is evidence regarding, for example, Alzheimer’s disease and Wilson’s disease), probably because of its involvement in the production of reactive oxygen species [2]. Because of its biological importance, fluorescent chemosensors that are able to monitor Cu^2+^ in living cells have attracted much attention in recent years [3,4,5,6,7,8].

Due to its high sensitivity and selectivity, as well as its noninvasive nature, fluorescence is very often selected as a signal for chemical sensing events.

**Figure 1:**
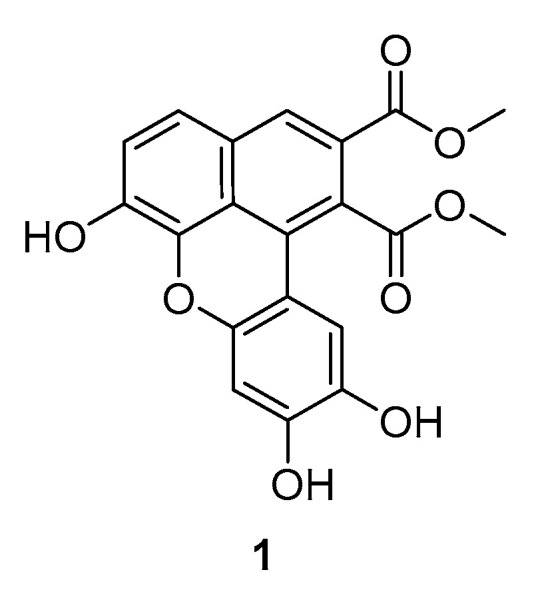
Structure of compound **1**.

Lately, our group has been involved in the synthesis, mediated by metals or enzymes, of bioinspired lignans and neolignans, including dimeric caffeic acid derivatives [9,10,11,12,13,14,15,16]. Notably, the oxidative coupling, mediated by manganese acetate (III), of caffeic acid esters or amides allowed us to obtain some natural and unnatural lignans with a planar and intensively fluorescent benzo[*k*,*l*]xanthene core, very rarely found in nature, exemplified by compound **1** (Figure 1) [17], which has recently been isolated from *Orobanche cernua* Loefling [18].

Because of the rarity of benzoxanthene lignans, their biological properties and possible applications are almost unexplored if we exclude some works in which these compounds have been proven as antifungal agents [19], anti-inflammatory agents [20], and useful antiproliferative compounds against different tumor cell lines, with antioxidant and antiangiogenic activities [17,21,22,23]. Some of these compounds were also subjected to STD-NMR measurements and molecular docking calculations; the obtained data indicate that benzoxanthene lignans interact with the DNA in two ways: the planar core intercalates between two base pairs in the DNA while the ester groups, which behave as flexible pendants, bind to the minor groove of the nucleic acid [17]. Moreover, the benzoxanthene lignans are strongly fluorescent both in solution and in the solid-state under UV-light at 366 nm, and this property may be of high interest for the design of new fluorescent probes for biomedical applications.

In this study, we investigated if the natural product **1**, selected among different previously reported compounds with a benzo[*k*,*l*]xanthene core, may be able to recognize metal cations that are involved in biological systems or that accumulate in the environment, namely Ag^+^, Cd^2+^, Co^2+^, Cu^2+^, Fe^3+^, Hg^2+^, K^+^, Mg^2+^, Na^+^, Ni^2+^, and Zn^2^^+^ [24,25].

## 2. Results and Discussion

The naturally occurring benzoxanthene lignan **1** was synthesized according to a biomimetic methodology based on the oxidative coupling of methyl caffeate, as previously reported [17]. The details are given in the experimental section.

The binding ability of compound **1** toward different metal cations (Na^+^, K^+^, Ag^+^, Hg^2+^, Cd^2+^, Co^2+^, Ni^2+^, Zn^2+^, Mg^2+^, Cu^2+^, and Fe^3+^) was studied by employing UV-Vis spectroscopy and fluorescence experiments. The progressive quenching of the fluorescence and any possible displacements of the absorption maxima in the UV-Vis spectrum were monitored as an indication of a structural variation of the substrate due to the interaction with the metal ion [26].

**Figure 2:**
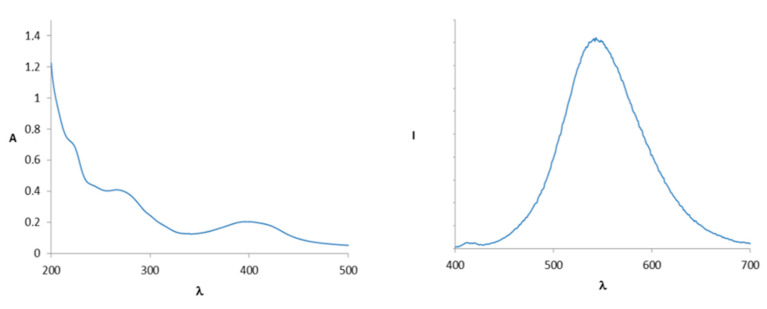
UV spectrum (**left**) and fluorescence spectrum (**right**) (excitation at 366 nm) of compound **1** (*c* = 1.72 × 10^−5^ M).

In Figure 2, we report the UV and fluorescence spectra (*λ* excitation at 366 nm) of compound **1** (1.72 × 10^−5^ M) in an ethanol-phosphate buffer (1:1).

**Figure 3:**
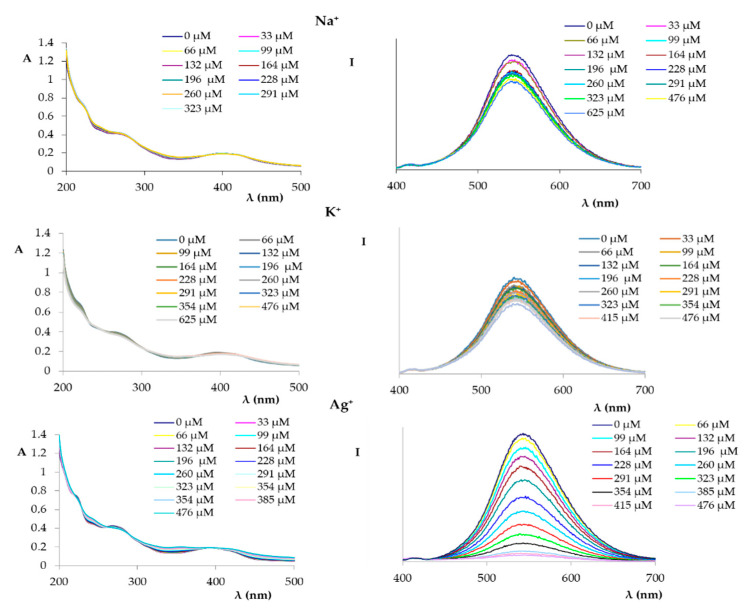
UV spectra (**left**) and fluorescence spectra (**right**) (excitation at 366 nm) of compound **1** (1.72 × 10^−5^ M in EtOH:phosphate buffer) titrated with an increasing amount of monovalent cations (1.0 × 10^−2^ M in phosphate buffer).

**Figure 4:**
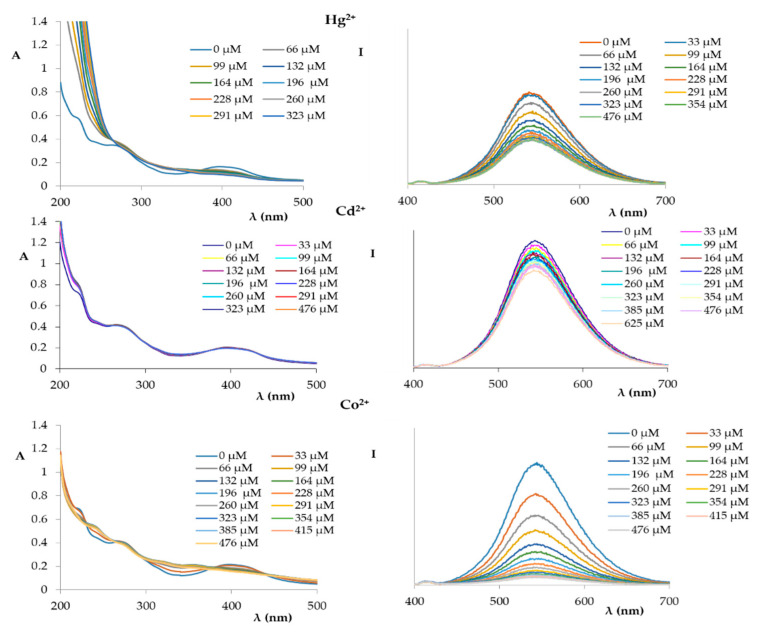
UV spectra (**left**) and fluorescence spectra (**right**) (excitation at 366 nm) of compound **1** (1.72 × 10^−5^ M in EtOH:phosphate buffer) titrated with an increasing amount of Hg^2+^, Cd^2+^, and Co^2+^ (1.0 × 10^−2^ M in phosphate buffer).

**Figure 5:**
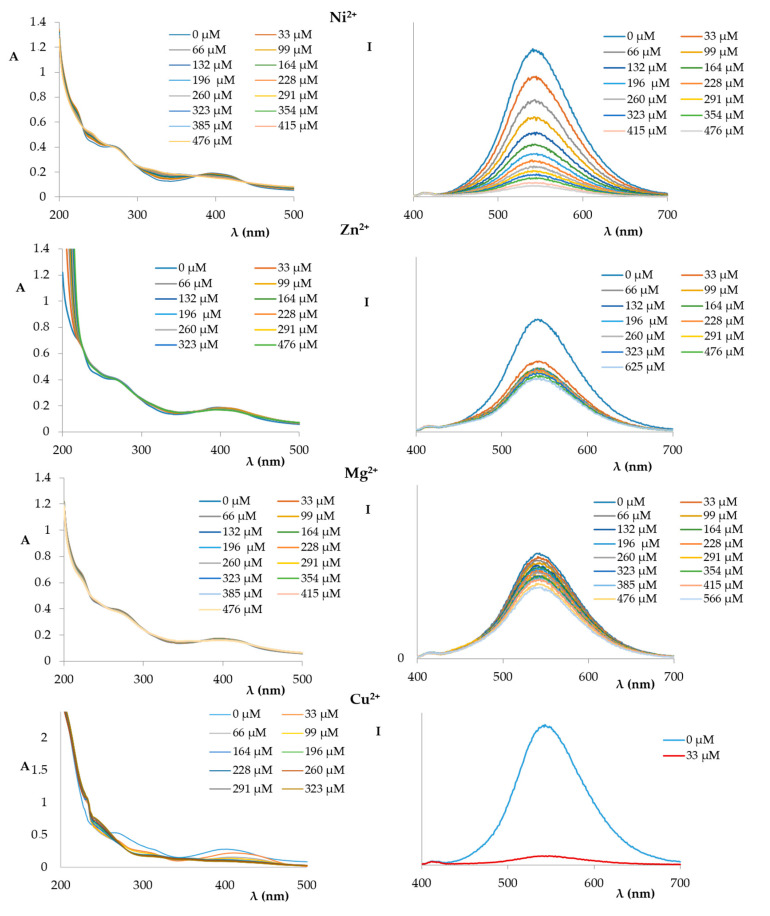
UV spectra (**left**) and fluorescence spectra (**right**) (excitation at 366 nm) of compound **1** (1.72 × 10^−5^ M in EtOH:phosphate buffer) titrated with an increasing amount of Ni^2+^, Zn^2+^, Mg^2+^, and Cu^2+^ (1.0 × 10^−2^ M in phosphate buffer).

**Figure 6:**
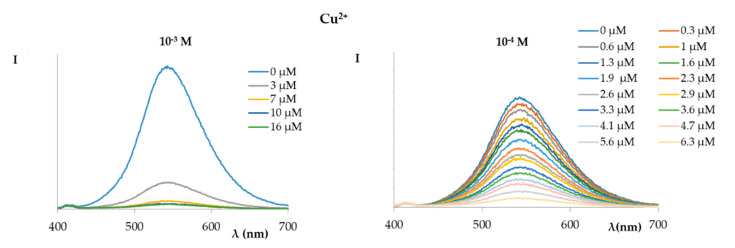
Fluorescence (excitation at 366 nm) spectra of compound **1** (1.72 × 10^−5^ M in EtOH:phosphate buffer) titrated with increasing amounts of a copper nitrate solution ((**left**) 1.0 × 10^−3^ M; (**right**) 1.0 × 10^−4^ M).

**Figure 7:**
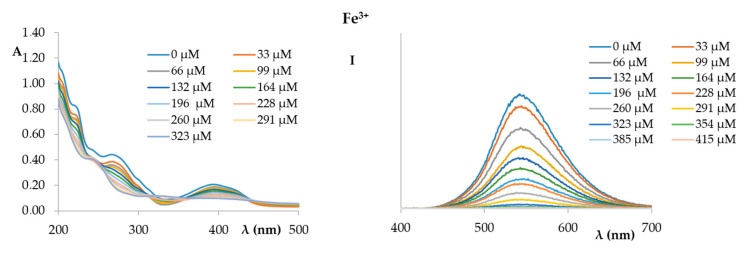
UV spectra (**left**) and fluorescence spectra (**right**) (excitation at 366 nm) of compound **1** (1.72 × 10^−5^ M in EtOH:phosphate buffer) titrated with an increasing amount of Fe^3+^ (1.0 × 10^−2^ M in phosphate buffer).

Subsequently, a series of solutions (10^−2^ M) of monovalent, bivalent, and trivalent ions, namely Na^+^, K^+^, Ag^+^, Hg^2+^, Cd^2+^, Co^2+^, Ni^2+^, Zn^2+^, Mg^2+^, Cu^2+^, and Fe^3+^, were prepared. The solutions were then used to record the UV and fluorescence spectra (Figure 3, Figure 4, Figure 5, Figure 6 and Figure 7) by successive additions of 10 μL aliquots to a solution of compound **1** in an ethanol-phosphate buffer 1:1 mixture (*c* = 1.72 × 10^−5^ M).

Figure 3 shows the UV and fluorescence spectra recorded in the presence of an increasing amount of the studied monovalent cations (Na^+^, K^+^, and Ag^+^).

As shown in Figure 3, the UV spectra did not show significant changes in the maxima absorption when compound **1** was titrated with each of the cations. However, some changes were observed in the fluorescence spectra. Notably, the spectra recorded in the presence of Na^+^ and K^+^ ions showed a maximum variation of 20 units initially, and subsequently, while increasing the concentration of the metal ions, no changes were observed. A different trend was observed when the same titration was carried out with Ag^+^ ions; in fact, a progressive decrease of the fluorescence intensity during the whole Ag^+^ titration was observed, which indicates an interaction between compound **1** and the Ag^+^ ions.

Both the UV and fluorescence spectra of compound **1** recorded in the presence of increasing amounts of divalent cations (Hg^2+^, Cd^2+^, Co^2+^, Ni^2+^, Zn^2+^, Mg^2+^, Cu^2+^) are shown in Figure 4 and Figure 5.

The spectra acquired by titration of compound **1** with Cd^2+^, Hg^2+^, Zn^2+^, and Mg^2+^ showed only an initial decrease of the fluorescence signal that subsequently reached a plateau, similar to the sodium and potassium ions.

However, a different trend was observed when the titration was made with Co^2+^ and Ni^2+^. In these cases, a progressive decrease in the intensity of the measured fluorescence was measured, similar to the silver ion.

A completely different trend was observed for the titration of the solution of compound **1** with a 1.0 × 10^−2^ M Cu^2+^ ion solution (Figure 5); in fact, the fluorescence was completely switched off after the first addition (10 µL) of the copper solution.

Therefore, further measures were carried out by lowering the concentration of Cu^2+^ ions. In Figure 6, the curves of the fluorimetric titrations of a solution of compound **1** (1.72 × 10^−5^ M) with increasing amounts of a solution of copper nitrate (1.0 × 10^−3^ M and 1.0 × 10^−4^ M) are shown. As can be observed, increasing the copper concentration via additions of a 10^−4^ M Cu^2+^ solution caused a linear reduction in the fluorescence intensity of the probe.

Figure 7 shows the UV and fluorescence spectra of compound **1** recorded in the presence of increasing amounts of Fe^3+^. Similar to what was observed for Ag^2+^, a progressive decrease in the intensity of the fluorescence of compound **1** was observed as a result of increasing additions of ferric ion.

The fluorescence of the benzoxanthene **1** in the presence of different metal ions was initially studied in the stoichiometric ratio of 1:1. From this study, it is evident that the intensity of fluorescence decreases after the addition of the different metal ions, and the extent of quenching depends on the different capacities of the metal ions to bind with the ligand. The addition of the studied metal ions other than Ag^+^, Cu^2+^, and Fe^3+^ only produced a minimal change in the intensity of emission at 543 nm.

**Figure 8:**
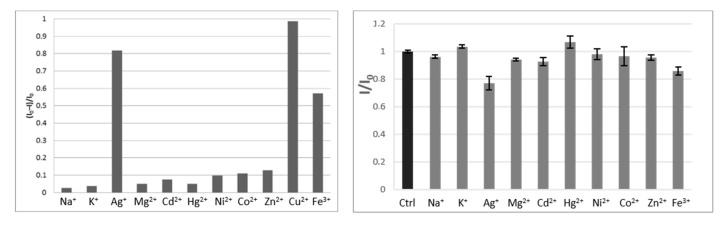
(**Left**) Comparison of the fluorescence intensity of compound **1** (1.72 × 10^−5^ M) with the studied metal ions (30 µM). (**Right**) Selectivity of compound **1** towards the studied cations. The concentration of Cu^2+^ was 1.4 µM; the concentration of the other metal ions was 40 µM. I_0_ and I are the fluorescence intensities of the mixtures of compound **1** and Cu^2+^ ions in the absence (Ctrl) and presence of other metals, respectively. Error bars represent standard deviations, as means of three experiments.

Figure 8 shows the ratio of fluorescence quenching for the different examined ions, calculated as (*I*_0_–*I*)/*I*_0_, which shows that Cu^2+^ led to a complete fluorescence quenching (98.7%), followed by Ag^+^ (82%) and Fe^3+^ (57%). Moreover, the natural probe **1** responded with high selectivity toward Cu^2+^ over the other metal ions, as confirmed by competing experiments (Figure 8, left). The fluorescence measurements of mixtures containing compound **1** in the presence of Cu^2+^ (1.4 µM) and one of the other metal ions (Na^+^, K^+^, Zn^2+^, Cd^2+^, Co^2+^, Ni^2+^, Hg^2+^, and Mg^2+^; each 40 µM) showed no relevant interference effect on the interaction of the Cu ion with the probe, even when the other cation was present in solution at a 30-fold higher concentration. Conversely, the experiments performed in the same conditions with Fe^3+^ and with Ag^+^ demonstrated a lower selectivity of probe **1** towards Cu ions, mostly in the presence of Ag ions.

Due to the high selectivity for Cu^2+^ and Ag^+^, subsequent studies were conducted only for these two ions.

The subsequent step was a study aimed at determining the stoichiometric ratio of metal–ligand and the binding affinities between the Cu^2+^ and Ag^+^ ions and compound **1**.

The association constants of the complexes (Cu^2+^/compound **1** and Ag^+^/compound **1**) were determined using UV and fluorescence titrations in solution followed by non-linear least-square regression. The titrations were also used to determine the stoichiometry of the obtained inclusion complexes. Different stoichiometries were tested (1:1, 1:2, 1:3; metals:compound **1**). The general method relying on the analysis of residual distribution in titration data fitting, considered the most reliable for establishing a proper binding model, was applied. After the refinement, the residuals with the 1:2 model for the Ag^+^ and the 1:3 model for the Cu^2+^ did not show a systematical trend, indicating that no relevant concentration of other complexes was formed under these experimental conditions. The association constant (*K*_β_) was evaluated when the concentration of compound **1** was fixed and the concentration of the metal was varied, as described in the experimental section. In the case of silver, the log*K*_β_ resulted in 6.589 ± 0.005, while for copper, we obtained a value of 13.265 ± 0.025, confirming the high selectivity of compound **1** for Cu^2+^.

**Figure 9:**
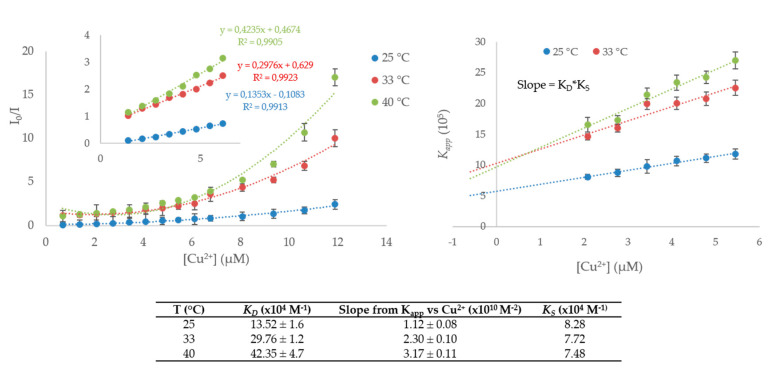
(**Left**) Stern–Volmer plots for the titration of compound **1** with Cu^2+^ at different temperatures. (**Right**) Modified Stern–Volmer plots. Each experiment was performed in triplicate. The obtained quenching constants are listed in the table.

Furthermore, based on this data, the fluorescence quenching process for probe **1** in the presence of copper ions was studied by the Stern–Volmer Equation [27],

(1) $$\frac{I_{0}}{I}=K_{SV}\left[Q\right]+1$$

where *I*_0_ and *I* are the fluorescence intensities of compound **1** before and after the successive additions of Cu^2+^ ions, respectively; [*Q*] stands for quencher concentration, namely the molar concentration of the Cu^2+^; and *K_SV_* is the Stern–Volmer quenching constant, which is an indicator of fluorescence quenching efficiency [28]. Figure 9 shows that the relative fluorescence (*I*_0_/*I*) linearly fitted with the Cu^2+^ ion concentration in the range of 1.5–5.5 µM (inset of Figure 9), while at higher concentrations, the plot shows an upward curvature, concave toward the *y*-axis. The characteristic feature of Stern–Volmer plots is indicative of a combination of static and dynamic quenching processes involving the fluorophore. In this case, the dependence of the fluorescence intensity upon quencher concentration is as follows:

(2) $$\frac{I_{0}}{I}=(1+K_{D}\left[Q\right])\times (1+K_{S}\left[Q\right])$$

where *K_D_* and *K_S_* are the dynamic and static quenching constants, respectively [29,30]. Moreover, from this equation, from the multiplication of the terms in parentheses, it is possible to obtain the following equation:

(3) $$K_{app}=\left[\frac{I_{0}}{I}-1\right]\times \frac{1}{\left[Q\right]}=(K_{D}+K_{S})+K_{D}K_{S}\left[Q\right]$$

A plot of apparent constant (*K_app_*) versus [Q] yields a straight line with an intercept of *K_D_* + *K_S_* and a slope of *K_S_* × *K_D_* (Figure 9, right). Furthermore, static and dynamic quenching can be distinguished by their different dependencies on temperature and viscosity, or preferably by lifetime measurements. We performed fluorimetric titration at three different temperatures, namely at 25, 33, and 40 °C (Supplementary Materials, Figure S1), and the results plotted in Figure 9 suggested that the change in the fluorescence intensity increases with temperature. Namely, higher temperatures yield faster diffusion, and hence larger amounts of collisional quenching occur. *K_D_* values were obtained by the slope of the regression curve in the linear range (Figure 9; Equation (1)), while *K_S_* values were estimated according to Equation (3), from the slope of the curve reported in Figure 9 (right) [30]. These results suggest that dynamic quenching has a considerable influence on the overall quenching processes and that the increase in temperature results in a lower quenching static constant.

Finally, the detection limit (LOD) was calculated according to the IUPAC recommendation as LOD = 3*σ*/slope, where *σ* is the standard deviation estimated by the fluorescence intensity of the blank, and the slope is obtained from a calibration curve for the fluorescence intensity against the Cu^2+^ ion concentration. Noteworthy, under these conditions, the LOD for the detection of Cu^2+^ ions in the presence of compound **1** is estimated to be 0.574 µM.

The reversibility of the Cu^2+^/compound **1** complex was evaluated, as shown in Figure S2 of Supplementary Materials. When the benzoxanthene **1** (1.70 × 10^−5^ M) was saturated by the addition of Cu^2+^ ions (0.2 equiv.), the fluorescence intensity was approximately halved; by adding a solution of EDTA (2.0 equiv.) to the mixture, a significant increase in the fluorescence intensity (almost up to the starting fluorescence value) was observed. These results demonstrate that the Cu^2+^ ion recognition of probe **1** is a reversible complexing process.

**Figure 10:**
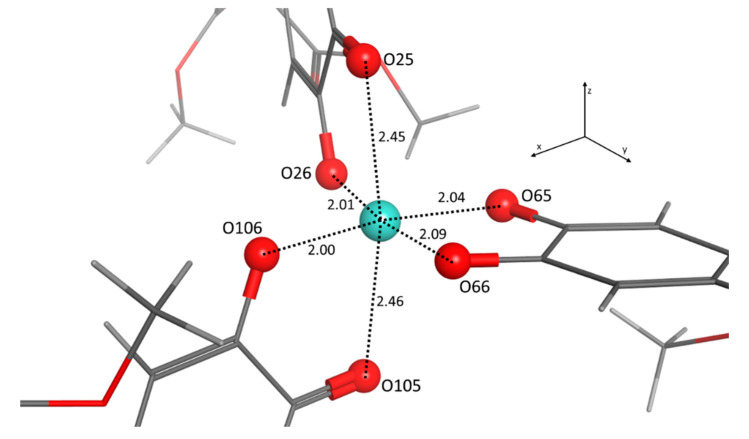
The geometry of the B3LYP/LANL2DZ optimized 1:3 Cu^2+^/compound **1** complex. Bond distances are showed in angstrom.

To support the experimental results, DFT calculations were performed on molecule **1** and Cu^2+^/compound **1** complex. The thermal complexation energy was studied for different stoichiometries (1:1, 1:2, 1:3; Cu^2+^:compound **1**). The data showed that the interaction energy (*E*_int_ = *E*_complex_ − *E***_1_** − *E*_Cu_^2+^) of the 1:3 complex was favorable among the three different complexes under study, resulting in −47.582 kcal/mol. The energies of the 1:2 and 1:1 complexes were −29.707 and −14.536 kcal/mol, respectively. The results of these energy calculations are in full agreement with the titration data, supporting the formation of the 1:3 complex. The geometry of the obtained complex is shown in Figure 10. In this geometry, the copper is complexed in a tetragonally elongated octahedral complex, where two oxygen (O65 and O66) of the same molecule of compound **1** and two oxygen atoms belonging to the other two molecules (O26 and O106) are lying in the *xy* plane, and the remaining O25 and O105 are located in the *z*-axis. The elongated octahedral is due to the Jahn–Teller effect that is often encountered in octahedral complexes of the transition metals and is very common in six-coordinate Cu^2+^ complexes [31,32]. According to the crystal field theory, the complexes of copper are principally square planar, with the odd electron occupying the d*x*^2^-*y*^2^ orbital; any addition ligands available would take up the fifth and sixth coordination positions, but because the d*z*^2^ always contains a pair of electrons, usually the fifth and sixth ligands (O25 and O105 in the case of Cu^2+^/compound **1** complex) will not be able to approach the copper as closely as the ligands in the plane [33].

The calculated Wiberg bond indexes for the complex are reported in Table 1, and as expected, they show the strongest interaction between the oxygen ligands in the *xy* plane and the metal. A less intense interaction is observed for the two oxygens in the *z*-axis. From the results of the secondary-order perturbation theory (SOPT) analysis of the Fock matrix in the natural bond orbital (NBO) basis, the single inter-complex contributions energies were also calculated. As expected from the obtained geometry of the complex, the strongest donor–acceptor interactions for the formation of the complex are the ones between the ion pairs of O26, O65, O66, and O106 with antibonding orbitals of the Cu^2+^. The highest energy donor–acceptor interactions are reported in Table 2. The energy gap between HOMO and LUMO for molecule **1** and Cu^2+^/compound **1** complex is found as 3.43 and 2.71 eV, respectively. TD-DFT calculation suggested that this transition corresponds to the first excited state. Thus, the obtained results indicate that the stabilized complex between molecule **1** and copper ion has a lower HOMO–LUMO energy gap when compared to compound **1** alone. The emission-quenching response of probe **1** was due to the strong interaction between copper and the hydroxylic groups as a result of the charge transfer process from the variation of the electronic structure of molecule **1** [34,35].

## 3. Materials and Methods

Materials were obtained from commercial suppliers and were used without further purification.

^1^H and ^13^C NMR spectra of compound **1** were run on a Varian VNMR-S spectrometer (Agilent, Milan, Italy) operating at 499.86 (^1^H) and 125.70 MHz (^13^C) and equipped with a gradient-enhanced reverse detection probe. NMR spectrometry experiments were performed using software supplied by the manufacturers and acquired at constant temperature (27 °C) in (CD_3_)_2_CO.

UV-Vis measurements were performed on a JASCO V630 spectrophotometer (JASCO Europe, Cremella (LC), Italy) using 1 cm quartz cuvette at 27 °C. The spectra were acquired in the range of 200–500 nm.

Fluorescence studies of compound **1** in the presence of the selected ions were carried out in a sealed cuvette at 27 °C in a Varian Cary Eclipse spectrophotometer (Agilent, Milan, Italy). Each sample in a cell of 1 cm path length was excited at 366 nm wavelength, and the emission scans were recorded from 400 nm to 700 nm; both the slits of excitation and emission were 10 nm. For UV-Vis and fluorimetric measurements, EtOH for spectroscopy and a 20 mM phosphate buffer (pH = 7.0), freshly prepared with double distilled water, were employed.

The association constants (*K*_β_) were determined by UV and fluorescence titrations, in conjunction with the non-linear least-square fit of the data to the models, using HypSpec software [36,37]. Several binding equilibria between compound **1** and the metals (Ag^+^ and Cu^2+^) were considered. The experimental data were either applied to single equilibrium model 1:1 or multiple binding equilibria models such as 1:1 + 1:2 + 1:3 (metals:compound **1**) models for best data fit with HypSpec software. The experimental data were tested against all the different types of binding models to establish a proper stoichiometry and association constant. The obtained values were almost identical for both UV and fluorescence titrations experiments.

### 3.1. Preparation of Methyl Ester of Caffeic Acid

Caffeic acid methyl ester was obtained by Fisher’s esterification. Caffeic acid (156.5 mg, 0.87 mmol) was treated with MeOH (35 mL) in the presence of a catalytic amount of concentrated H_2_SO_4_ (0.2 mL). The resulting mixture was stirred at reflux temperature for 4 h. The mixture was diluted with ethyl acetate (100 mL), and the pure product was quantitatively recovered from the organic phase by partitioning with water.

### 3.2. Preparation of Dimethyl 6,9,10-Trihydroxybenzo[k,l]Xanthene-1,2-Dicarboxylate *(**1**)*

The benzoxanthene **1** was synthesized according to the previously reported biomimetic methodology. Briefly, caffeic acid methyl ester (100.2 mg, 0.26 mmol) was solubilized in CHCl_3_ (18.5 mL), and the solution was mixed with an excess of Mn (OAc)_3_ (278.8 mg, 1.04 mmol). The reaction mixture was stirred at room temperature for 2 h. The reaction was quenched by the addition of a saturated ascorbic acid solution (15 mL), and the mixture was partitioned. The aqueous phase was partitioned with CH_2_Cl_2_ (3 × 15 mL), the combined organic layer was dried over anhydrous Na_2_SO_4_, and the solvent was evaporated under vacuum. The residue was purified by chromatographic column on Chromabond silica diol (45 µm; Macherey-Nagel) using a gradient of MeOH in CH_2_Cl_2_ (from 1% to 5%). UV (EtOH:phosphate buffer 1:1) *λ*_max_ (log *ε*) 272 (4.29), 396 (4.01) nm. ^1^H and ^13^C NMR data of compound **1** are in perfect agreement with those previously reported in the literature [17,21].

### 3.3. Preparation of UV-Vis and Fluorometric Titration Solutions

Stock solutions (0.01 M) of nitrate and chloride salts of Ag^+^, Cd^2+^, Co^2+^, Cu^2+^, Fe^3+^, Hg^2+^, K^+^, Mg^2+^, Na^+^, Ni^2+^, and Zn^2+^ in phosphate buffer (20 mM, pH = 7.0) were prepared. A probe stock solution of compound **1** (1.72 × 10^−5^ M) was prepared using EtOH-buffer (1:1). A solution of compound **1** (3 mL) of the probe stock solution was put into a test tube and titrated with the successive addition (10 µL) of each ion’s stock solution. The spectrofluorimetric measurements were performed at 25, 33, and 40 °C, and the data were elaborated according to the Stern–Volmer equation (Equations (1)–(3)).

For competing experiments, a 1.70 × 10^−5^ M solution of compound **1** was used to record fluorimetric measurements in the presence of Cu^2+^ (1.4 × 10^−6^ M; control) and one of the other metals under study (Na^+^, K^+^, Ag^+^, Cd^2+^, Co^2+^, Hg^2+^, Mg^2+^, Ni^2+^, Zn^2+^, and Fe^3+^; 40 × 10^−6^ M).

### 3.4. Theoretical Calculations

Molecules were drawn in Marvin Sketch (18.24, ChemAxon Ltd., Budapest, Hungary), and 3D-structures were initially generated with the Merck molecular force field (MMFF94) and then optimized at a semiempirical level using the parameterized model number 6 (PM6) Hamiltonian as implemented in the MOPAC package (MOPAC2016 v. 18.151, Stewart Computational Chemistry, Colorado Springs, CO, USA) [38], before the DFT calculations.

The DFT calculations were performed using the Gaussian 16 software (Gaussian, Inc., Wallingford, CT, USA) with B3LYP and LANL2DZ basis sets [39].

## 4. Conclusions

In conclusion, we report a benzo[*k*,*l*]xanthene derivative, molecule **1**, which is a selective and sensitive turn-off chemosensor that recognizes the Cu^2+^ ion explicitly in an EtOH-phosphate buffer (1:1) solution by fluorescence spectroscopy. Compound **1** was selected among different previously reported compounds with a benzo[*k*,*l*]xanthene core, and its complexation ability toward different metal cations that are involved in biological systems or that accumulate in the environment (such as Ag^+^, Cd^2+^, Co^2+^, Cu^2+^, Fe^3+^, Hg^2+^, K^+^, Mg^2+^, Na^+^, Ni^2+^, and Zn^2+^) was studied. The natural benzoxanthene **1** was shown to respond with high selectivity toward Cu^2+^ over the other metal ions. Among all of the studied ions, the molecule showed an ion concentration-dependent decreasing fluorescence for Ag^+^ and, at a lower concentration, for Cu^2+^. For this latter cation, a LOD of 0.574 µM was found. The calculated log*K*_β_ resulted in 6.589 for Ag^+^ and 13.265 for Cu^2+^. Theoretical DFT calculation showed that the geometry of the Cu^2+^/compound **1** complex is a tetragonally elongated octahedral and supported the formation of a 1:3 stoichiometry. Fluorescence experiments demonstrate that compound **1** may have application as a fluorescent probe for detecting Cu^2+^ over a wide range of other cations without interferences.

## Figures and Tables

**Table 1:** Calculated Wiberg bond indexes between the indicated oxygen atoms and the Cu^2+^.

Atom	O25	O26	O65	O66	O105	O106
Wiberg bond index	0.1263	0.2850	0.2300	0.2717	0.1283	0.2850

**Table 2:** Secondary orbital interactions for Cu^2+^/compound **1** complex.

Donor NBO	Acceptor NBO	Energy (kcal/mol)
LP O25	LP* Cu121	3.93
LP O25	LP* Cu121	3.81
LP O26	LP* Cu121	13.86
LP O26	LP* Cu121	12.08
LP O65	LP* Cu121	11.28
LP O65	LP* Cu121	10.14
LP O66	LP* Cu121	13.21
LP O66	LP* Cu121	9.71
LP O105	LP* Cu121	4.24
LP O105	LP* Cu121	3.89
LP O106	LP* Cu121	12.87
LP O106	LP* Cu121	12.49

## Supplementary Materials

Supplementary Materials can be found at https://www.mdpi.com/1422-0067/21/18/6933/s1.
