# Supplementary material for: A Rare Natural Benzo[k,l]xanthene as a Turn-Off Fluorescent Sensor for Cu2+ Ion
Source: Int J Mol Sci. 2020 Sep 21;21(18):6933. doi: 10.3390/ijms21186933 (PMC7555586; doi:10.3390/ijms21186933)
Supplement: Supplementary file 1 [file ijms-21-06933-s001.pdf]

International Journal of *Molecular Sciences*

MDPI

Supplementary Materials

A Rare Natural Benzo[*k*,*l*]xanthene as a Turn-Off Fluorescent Sensor for Cu^{2+} Ion

Giuseppe Floresta [1], Nunzio Cardullo [2,*], Carmela Spatafora [2,†], Antonio Rescifina [1,*] and Corrado Tringali [2]

1 Department of Drug Sciences, University of Catania, Viale A. Doria 6, 95125-Catania, Italy; giuseppe.floresta@unict.it

2 Department of Chemical Sciences, University of Catania, Viale A. Doria 6, 95125-Catania, Italy; cspatafo@unict.it (C.S.); ctringali@unict.it (C.T.)

* Correspondence: ncardullo@unict.it (N.C.); arescifina@unict.it (A.R.)

† Dedicated to Professor Carmela Spatafora, a friend, colleague, and distinguished scientist, who prematurely passed away during the preparation of the paper.

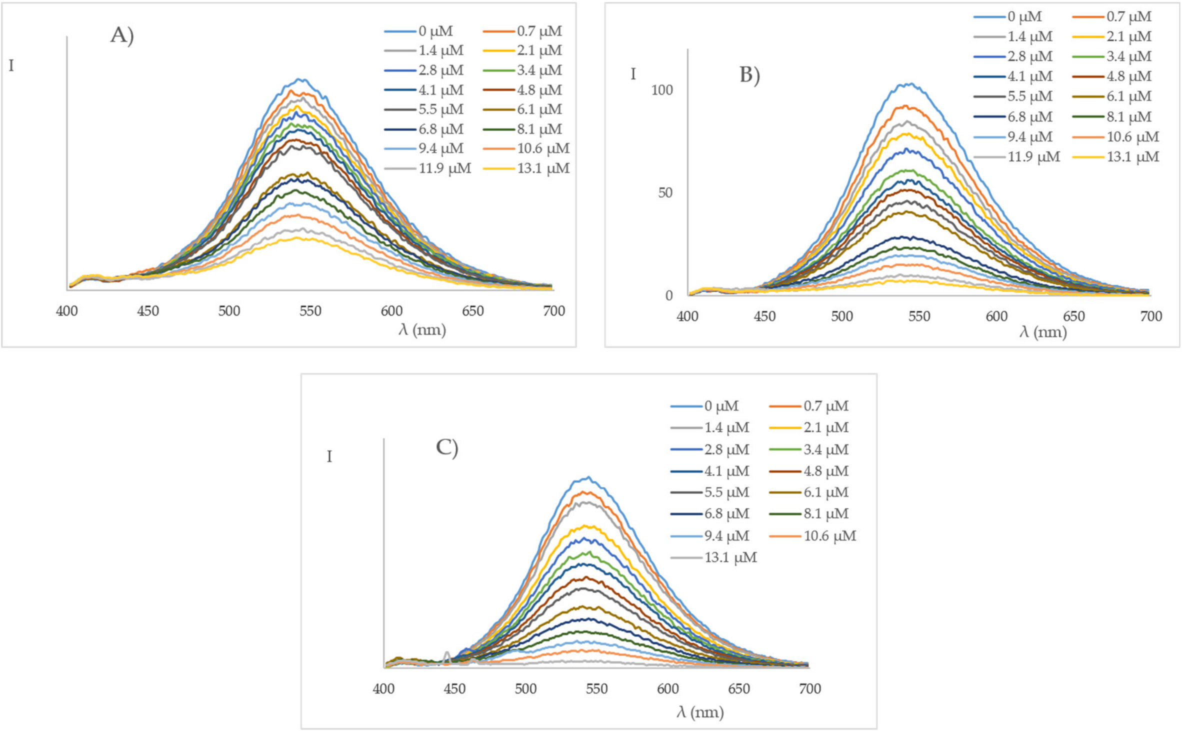

Figure S1. Representative fluorescence spectra of **1** (1.89×10^{-5} M; EtOH: phosphate buffer, pH = 7.0) in the presence of different aliquots of Cu^{2+} (2.1×10^{-4} M in phosphate buffer) at: (**A**) 25 °C, (**B**) 33 °C, (**C**) 40 °C.

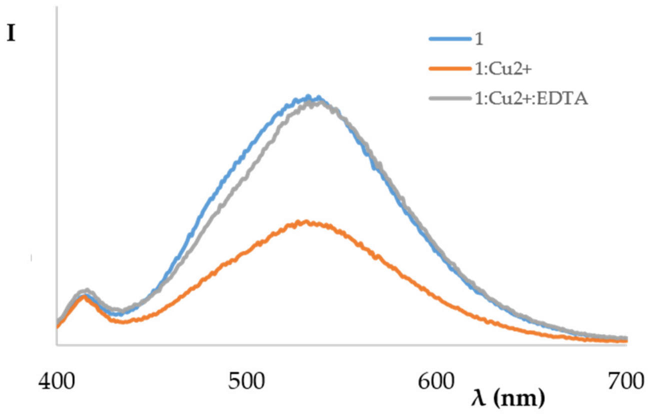

Figure S2. Fluorescence spectra of **1** (1.69×10^{-5} M; EtOH: phosphate buffer, pH = 7.0) upon addition of Cu^{2+} (0.5 equiv.) and then EDTA (2 equiv.).

© 2020 by the authors. Licensee MDPI, Basel, Switzerland. This article is an open access article distributed under the terms and conditions of the Creative Commons Attribution (CC BY) license (http://creativecommons.org/licenses/by/4.0/).
